# Life history responses of two ephemeral plant species to increased precipitation and nitrogen in the Gurbantunggut Desert

**DOI:** 10.7717/peerj.6158

**Published:** 2019-01-11

**Authors:** Yanfeng Chen, Lingwei Zhang, Xiang Shi, Huiliang Liu, Daoyuan Zhang

**Affiliations:** 1 Key Laboratory of Biogeography and Bioresource in Arid Land, Xinjiang Institute of Ecology and Geography, Chinese Academy of Sciences, Urümqi, Xinjiang, China; 2 University of Chinese Academy of Sciences, Beijing, China; 3 College of Grassland and Environment Sciences, Xinjiang Agricultural University, Urümqi, Xinjiang, China; 4 College of Agriculture, Shihezi University, Shihezi, Xinjiang, China; 5 Yili Botanical Garden, Xinjiang Institute of Ecology and Geography, Xinyuan, Xinjiang, China; 6 Turpan Eremophytes Botanical Garden, Chinese Academy of Sciences, Turpan, Xinjiang, China

**Keywords:** Climate change, Ephemeral plant, *Eremopyrum distans*, Gurbantunggut Desert, Increased precipitation, Interaction, *Nepeta micrantha*, Nitrogen deposition

## Abstract

Precipitation change and nitrogen deposition are not only hot topics of current global change but also the main environmental factors affecting plant growth in desert ecosystems. Thus, we performed an experiment of increased precipitation, nitrogen, and precipitation plus nitrogen on the ephemeral annual species *Nepeta micrantha* and *Eremopyrum distans* in the Gurbantunggut Desert. We aimed to determine the life history responses of *N. micrantha* and *E. distans* to environment changes, and the germination percentage of the offspring (seeds) was also tested in the laboratory. The results showed that increased nitrogen and precipitation plus nitrogen increased the growth of both plant species, whereas increased precipitation inhibited the growth of *N. micrantha* but increased the growth of *E. distans*. This differential response of these two species to precipitation and nitrogen also affected the germination of their offspring. In response to increased nitrogen and precipitation plus nitrogen, the germination percentage of the offspring produced by two species decreased in conjunction with the plants exhibiting high reproduction, which may prevent overcrowding during the following year; however, the *N. micrantha* plants produced more nondormant offspring in conjunction with low reproduction under relatively greater amounts of precipitation, and *N. micrantha* offspring could occupy their habitat via rapid germination in suitable environments. Therefore, with increased precipitation and nitrogen deposition, these differences in offspring dormancy may affect their ecological niche in the community.

## Introduction

Anthropogenic activity and climate change have a continuing effect on global and regional environments, including widespread changes in precipitation and nitrogen deposition (Stocker et al., 2013). Plants are an important component of terrestrial ecosystems and play an important role in their regulation. Therefore, understanding plant responses to precipitation and nitrogen deposition is important for maintaining ecosystem stability (Cabaço & Rui, 2012). Especially in arid and semiarid areas where environmental conditions are extremely harsh, slight disturbances are likely to cause a severe changes in plant growth (Knapp & Smith, 2001; Huxman et al., 2004; Diffenbaugh, Giorgi & Pal, 2008). Moisture is the most important limiting factor for plant growth in arid and semiarid regions, and precipitation is the primary source of soil moisture (Dube & Pickup, 2001; Cheng et al., 2006). In the Gurbantunggut Desert of northwestern China, the annual precipitation is predicted to increase by 30% during the next 30 years (Bai et al., 2008; Liu et al., 2010; Huang et al., 2017). Nitrogen deposition from the nearest city to the study area is 35.4 kg ha^−1^ yr^−1^, and the nitrogen deposition in the Gurbantunggut Desert is predicted to double in the future (Liu et al., 2013; Xu et al., 2015). Therefore, researching plant responses to precipitation and nitrogen deposition in this desert ecosystem is highly necessary to adapt to climate change.

Precipitation is a key climate factor that affects plant growth, especially in desert ecosystems (Hsu, Powell & Adler, 2012; Beer et al., 2010). During the seedling stage, seedling emergence of desert annual plants increased significantly and exhibited faster germination time with increased precipitation in the Mu Us Desert (Zheng et al., 2005), but the contrary was the case with the decreasing of soil moisture (Liu et al., 2018). During the growth stage, increased precipitation not only promoted the production of new leaves and branches (Zhang et al., 2015), but also increased biomass accumulation in desert annual species (Shemtov & Gutterman, 2003). But the promotion of precipitation often intensify competition among species. Small-stature plants can be overshadowed by taller plants and the ground cover by small-stature plants decreases with increased precipitation (Harpole, Potts & Suding, 2007; Zavaleta et al., 2003). The moisture requirement for non-native annual grasses is typically lower than that of native plants leading to an increased advantage in non-native annual grass competition in the Mojave Deserts (Horn, Nettles & Clair, 2015). Thus, desert annual herbs respond very rapidly to precipitation changes throughout the whole growth period and species show differential responses to increased precipitation in the community.

Although desert plants are primarily limited by precipitation, previous studies have indicated the potential for co-limitation by nitrogen in desert ecosystems (Hall et al., 2011). Hence, nitrogen fertilization is commonly used to improve plant growth in semiarid/arid ecosystems (Frink, Waggoner & Ausubel, 1999). In the Mojave Desert, soil nitrogen addition increased the dominance of alien annual plants, but inhibited the growth of natives (Jordan, 1996). At the Sevilleta, the cover of two dominant species (black grama and blue grama) is not changing directionally by changes in nitrogen deposition, but are likely caused by other factors (Baez et al., 2007). In the Chihuahuan Desert grassland, nitrogen limitation only becomes evident on plant growth following periods of above average precipitation (Ladwig et al., 2012). Therefore, these different responses of desert plants to nitrogen likely derive from the variations in species specificity and other factors, especially in precipitation regime.

Water is not only a material for photosynthesis (Maberly, 2014), but also a solvent for nitrogen (Gebauer & Ehleringer, 2000; Lloret, Casanovas & Penuelas, 2010). Similarly, the effects of precipitation plus nitrogen on plants have been reported largely in terms of plant richness (McHugh et al., 2017; Simkin et al., 2016), diversity (Bobbink et al., 2010; McHugh et al., 2017), community coverage (McHugh et al., 2017), community biomass (Xu et al., 2018), and plant-plant interactions (Wang et al., 2016a; Wang et al., 2016b). However, the effects of precipitation plus nitrogen on whole life history have rarely been reported. The only reports to date have focused on certain aspects of plant life history; for example, the nitrogen plus precipitation significantly advanced the onset of the flowering and fruiting times (Huang, Li & Li, 2018) and was shown to increase root growth (Zhang et al., 2017), leaf number (Barker et al., 2006; Huang, Li & Li, 2018), seed yield (Altenbach et al., 2003; Fuertes-Mendizabal et al., 2010), and biomass accumulation (Al-Solaimani et al., 2017). No research on the entire life history has been performed with respect to the effect of precipitation plus nitrogen deposition.

Ephemeral plants are a unique group of plants and widely distributed in the Gurbantunggut Desert (Lu et al., 2014). Ephemeral plant coverage can reach 40% of this area in May, while coverage by shrubs and long-living herbs on the surface of the dunes reaches less than 10% (Wang et al., 2003). Therefore, ephemeral plants are the primary contributors to the stabilization of the sand surface during early spring. By utilizing winter snowmelt and spring rainfall, ephemeral plants complete their life cycle quickly (Lu et al., 2016). Compared with other annual and perennial herbs, ephemeral plants grow more quickly and exhibit greater light use efficiency, and they allocate a greater percentage of their biomass to reproduction (Yuan & Tang, 2010). Thus, ephemeral plants are extremely sensitive to climate change. Previous research on the effects of precipitation and nitrogen on ephemeral plants has focused primarily on plant survival, biomass accumulation (Lu et al., 2014) and nutrient use (Wang et al., 2016a; Wang et al., 2016b). The information about plant responses to climate change throughout the entire life history is lacking.

The importance of root traits as drivers of ecosystem function has attracted widespread concern (Bardgett, Mommer & De Vries, 2014). In arid-semiarid ecosystems, the root morphology can influence the ability of the plants to regulate their own growth in response to environmental change (Ogle & Reynolds, 2004; Shen, Reynolds & Hui, 2010; Griffin-Nolan et al., 2018). For example, Paz, Pinedagarcía & Pinzónpérez (2015) reported that 23 species exhibit wide variation in their root morphology, which ultimately governs plant mass by absorbing water and nutrients. Two common annual ephemeral plants in the Gurbantunggut Desert during early spring include *Eremopyrum distans* and *Nepeta micrantha* (Wang et al., 2010; Ding et al., 2016). *E. distans* is a member of the Gramineae and has a fibrous root system (Wang et al., 2010). *N. micrantha* is a member of the Labiatae and has a taproot system (field observations by the first author from 2014 to 2016). Therefore, the root morphology forms of *E. distans* and *N. micrantha* are significantly different. On the basis of the sensitivity response of ephemeral plants to environmental change, the effects of roots in regulate the response of plant to environmental change and the significant differences in root morphology of *E. distans* and *N. micrantha*, we hypothesized that the life histories of *E. distans* and *N. micrantha* differ in response to increased precipitation and nitrogen and precipitation plus nitrogen in the field. To test this hypothesis, we compared the phenology, morphological traits, biomass accumulation and allocation, and offspring (seed) germination of *E. distans* and *N. micrantha* plants during the life history.

## Materials and Methods

### Study site

The study site is located on the southern edge of the Gurbantunggut Desert, which is the second largest desert in China (Wang et al., 2004). This desert has a typical temperate continental climate with hot summers and cold winters; the highest annual temperature is 42.6 °C, the lowest annual temperature is −41.6 °C, and the annual precipitation is generally 70–100 mm. The evapotranspiration can reach approximately 2,000 mm, which is 20–30 times the precipitation (Ji, Ye & Wei, 2000). During the winter, the Gurbantunggut Desert has a stable snow layer and a maximum snow depth of more than 20 cm, which lasts from three to five months (Zhou, Zhou & Dai, 2009; Li et al., 2010). During the early spring, the snow melts rapidly and provides sufficient water for the growth of ephemeral plants (Wang et al., 2003).

### Experimental design

#### Increased precipitation and nitrogen treatments

To simulate the current precipitation amounts in the Gurbantunggut Desert as well as the future and extreme amounts expected, an experiment was established to simulate precipitation increases of +0%, +30%, and +50% based on empirical precipitation data from the study area during the spring and summer. Owing to the rapid development of the economy, the Gurbantunggut Desert has been surrounded by farmland and factories; therefore, it receives a large exogenous input of nitrogen. The nitrogen deposition from a nearby city (Urumqi) has reached 35.4 kg ha^−1^ yr^−1^(Wei, 2015). According to Galloway et al. (2008), global nitrogen deposition will increase rapidly doubling the amount recorded in 1990 within 30 years. Thus, we simulated three N deposition levels in the experiment: N0 (control treatment, no additional nitrogen input, the value is the current actual nitrogen input), N1 (the current deposition rate in a polluted area, 3 g N m^−2^ yr^−1^), and N2 (the predicted future rate of 6 g N m^−2^ yr^−1^). The field experiment consisted of three levels of precipitation (+0%, +30%, and +50%; CK, W1, and W2, respectively), three levels of N (0, 3, and 6 g N m^−2^ yr^−1^; CK, N1, and N2, respectively), and the precipitation plus nitrogen (W1N1 and W2N1). A total of seven treatments (CK, W1, W2, N1, N2, W1N1, and W2N1) were set up with eight replicates for per treatment for a total of 56 plots. The plots were distributed at the bottom of the flat dunes, and the size of each plot was 1 m ×1 m and encompassed 12 plants. To avoid interference, we removed other species from the plots in November 2016 and again in April 2017. We installed plastic film around the plots to avoid affecting the environment. Furthermore, in order to collect rainfall for the increased precipitation experiments, four precipitation collection vessels were installed in the field. Each precipitation collection vessel was assembled from plastic boards (4 m ×4 m) and iron poles. Each board was installed at an angle of 30° from the ground to collect precipitation, and one bucket was placed on the lower corner of the board.

On September 1, 2014, we installed a weather and soil moisture monitoring device (Caipos GmbH, Schiller Strasse Gleisdorf, Austria) to monitor the precipitation, temperature, and soil moisture. When a precipitation event occurred, the monitoring device collected data from wireless sensors, uploading the measurements directly to a web-based platform every hour. We conducted an increased precipitation experiment on the second day after each natural precipitation events (including some snow fall in early spring). We used a watering can to apply rainwater on the plots designated for testing increased precipitation.

With respect to nitrogen applications, we referred to the methods of Zhou et al.’s (2011) and applied nitrogen twice per year in late March (snowmelt) and late October (before snowfall). When the nitrogen was applied, NH_4_NO_3_ and NH_4_Cl were dissolved in the water at NH_4_:NO_3_ = 2:1, and the solution was manually applied evenly on the sample surface area; the same amount of water was applied on the control soils.

### Experimental material

We sowed seeds in the designated plots in the beginning of October, 2016. One hundred *E. distans* and *N. micrantha* seeds were evenly sown each 1 m ×1 m plot. On March 10, 2017, 12 of the largest plants in each 1 m ×1 m plot were marked, after which excess seedlings and other species were removed.

### Measurements and sampling

#### Phenology

We followed the methods of Lu et al. (2014) and observed the experimental plots every 3 days from germination until death, and we recorded the emergence date (the number of days until all the seeds in each treatment had emerged), flowering date (the number of days until all the plants in each treatment had flowered), fruiting date (the number of days until the occurrence of the first green fruit on all the plants in each treatment) and maturation date (the number of days until the first fruits of all the individuals plants in each treatment had turned a khaki color and were ready to disperse naturally) were determined. The post germination life span (the interval from the emergence of the first individual to the death of the last individual in each treatment) was then determined.

#### Plant traits

When the plants reached maturity, their traits (plant height; leaf area; root length; and the numbers of flowers, fruits, and seeds) were measured. We measured the leaf area of fresh leaves using a LI-COR 3000 leaf area meter directly. However, we measured the leaf area of the curling leaves after they were rehydrated. The total leaf area was calculated as the area of the fresh leaves plus that of the curled leaves. The root of *N. micrantha* has a tap root system and consists mainly of the main root and a small number of lateral roots that are growing on the main root, so, we measured the length of main root to represent the root length. However, root of *E. distans* has a fibrous root system, and it consists of many fibrous roots. Therefore, the root length of *E. distans* is expressed by taking the average of the fibrous roots.

#### Dry mass accumulation and allocation

After the plant traits were measured, the plants in all the treatments were harvested and separated into roots, stems, leaves, and reproductive organs (flowers, fruits, and seeds), and the roots were carefully washed free of soil. We harvested the seeds or fruits at maturity, i.e., when the fruits were dry, yellow, and dehiscing. As a result of the different precipitation and nitrogen treatments, the seeds or fruits reached maturity at different times. Thus, we harvested the plants as soon as their seeds or fruits had turned yellow. Various organs (fruits, leaves, stems, and roots) from the plants were weighed separately using a Sartorius BS210S electronic balance (0.0001 g) after they were dried at 75 °C for 48 h. The total biomass was calculated as the masses of the various organs (roots, stems, leaves, fruits and seeds) per plant. The biomass allocation of the roots, stems, leaves, and reproductive organs (flowers, fruits, and seeds) was expressed as a percentage as follows:

\begin{eqnarray*}\text{Root} \mathrm{p}\text{ercentage}=(\text{roots mass})/(\text{total mass})\times 100\text{%}; \end{eqnarray*}

\begin{eqnarray*}\text{Stem} \mathrm{p}\text{ercentage}=(\text{stems mass})/(\text{total mass})\times 100\text{%}; \end{eqnarray*}

\begin{eqnarray*}\text{Leaf} \mathrm{p}\text{ercentage}=(\text{leaves mass})/(\text{total mass})\times 100\text{%}; \end{eqnarray*}

\begin{eqnarray*}\text{Reproductive} \mathrm{p}\text{ercentage}=(\text{reproductive mass})/(\text{total mass})\times 100\text{%}. \end{eqnarray*}

#### Offspring(seed) germination

Mature fruits from the different treatments were collected on May 21 and June 1, 2017. We removed the shells and retained the seeds from *E. distans* and *N. micrantha* for the germination experiment. With respect to *E. distans*, germination tests were conducted at an alternating temperature regimen of 25 °C/10 °C (12 h of light/12 h of darkness) starting on June 20, 2017. With respect to *N. micrantha,* germination tests were conducted at a constant temperature regime of 14 °C (24 h of darkness) starting on June 20, 2017 (these temperature and light settings were determined via another germination experiment on *E. distans* and *N. micrantha*; Y Chen, 2019, unpublished data). Twenty-five seeds were placed on two layers of Whatman No. 1 filter paper that was moistened with 3 mL of distilled water in each of the four 7 cm Petri dishes. Water was added as needed to keep the filter paper moist during the test period. A seed was considered to have germinated when its radicle had emerged. The germinated seeds were counted and removed daily for 30 days. The final percentage of germination (FPG) was estimated as follows: FPG = GN∕SN, where GN is the total number of germinated seeds and SN is the number of viable seeds. If the intact seeds did not germinate after the germination experiment, their viability was tested by using a 1% triphenyltetrazolium chloride TTC solution (Baskin & Baskin, 2014).

### Statistical analyses

Data collation and analysis were performed using Microsoft Excel and SPSS 16.0 (SPSS Inc., Chicago, Illinois, USA) software, respectively, and figures were constructed with Origin 8.0 software (Origin Lab, Northampton, MA). Plant traits (plant height, leaf area, leaf number, root length and seed number), dry mass accumulation and allocation (total dry mass and proportion allocated to roots, stems, leaves and reproductive organs) and the final percentage of germination were analyzed as dependent variables, and increased precipitation, nitrogen and precipitation plus nitrogen were considered as fixed effects with a one-way ANOVA. Tukey’s test was performed for multiple comparisons to determine significant differences among treatments, and we performed a Bonferroni correction to avoid type I error problems. The allocation results for the roots, stems, leaves and reproductive organs (percentage data) were transformed (log10) before a one-way ANOVA was conducted. Pearson correlations were calculated for data involving the phenology, plant traits, and biomass accumulation and allocation from all treatments.

## Results

### Precipitation and temperature

**Figure 1:**
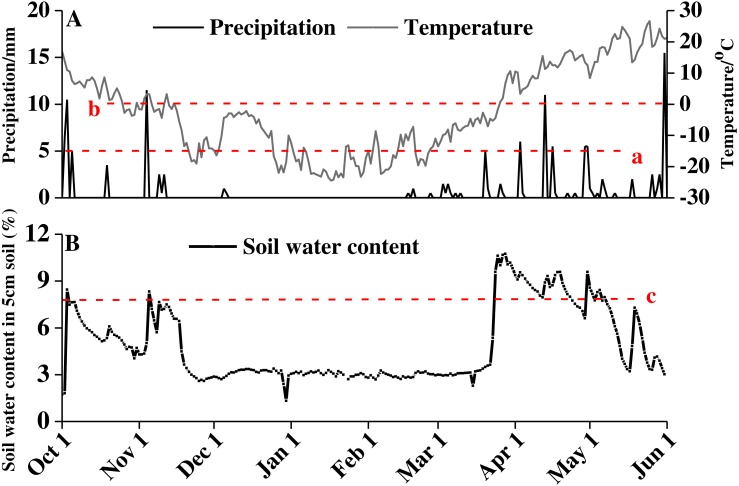
Precipitation, temperature (A), and soil water content (B) of study area from October 1, 2016 to June 1, 2017. Red lowercase letter a indicates that precipitation amount exceed 5 cm, red lowercase letter b indicates precipitation amount exceed 10 cm, and red lowercase letter c indicates soil water content above 8%.

From October 2016 to June 2017, the average daily temperature decreased first and then increased, with a minimum of −24.48 °C on January 17, 2017 and a high of 26.6 °C on May 25, 2017 (Fig. 1A). The snow thickness was 20.23 ± 0.40 cm on February 16, 2017, and the snow began to melt on March 20, 2017. Water from the snowmelt caused a sharp increase in soil moisture at the end of March. The soil moisture content remained at approximately 8% from late March until late April (Fig. 1B).

### Phenology

Increased precipitation (W1 and W2), nitrogen (N1 and N2), and precipitation plus nitrogen (W1N1 and W2N1) delayed the flowering date, fruiting date, and withering date and prolonged the life cycles of *N. micrantha* and *E. distans* significantly (*P* < 0.01) (Table 1).

**Table 1:** Effects of increased precipitation and nitrogen and precipitation plus nitrogen treatments on the phenology of *Nepeta micrantha* and *Eremopyum orientale* (mean ± 1s.e).

	**Phenology**	**CK**	**W1**	**W2**	**N1**	**N2**	**W1N1**	**W2N1**
***Nepeta micrantha***	**Emergence date**	16 April	16 April	16 April	16 April	16 April	16 April	16 April
	**Four-leaf rosette date**	20 April	20 April	20 April	19 April	20 April	19 April	19 April
	**Flowering date**	6 May	6 May	6 May	9 May	5 May	8 May	7 May
	**Fruiting date**	14 May	15 May	15 May	15 May	14 May	15 May	15 May
	**Withering date**	24 May	25 May	25 May	25 May	24 May	25 May	25 May
	**Life cycle (d)**	47.80 ± 0.006^b^	48.80 ± 0.467^ab^	49.40 ± 0.340^a^	49.30 ± 0.300^a^	48.40 ± 0.340^ab^	48.70 ± 0.396^ab^	49.20 ± 0.442^a^
***Eremopyrum orientale***	**Emergence date**	10 Oct	10 Oct	10 Oct	10 Oct	10 Oct	10 Oct	10 Oct
	**Four-leaf rosette date**	23 April	23 April	23 April	23 April	23April	23April	23 April
	**Flowering date**	∼	∼	∼	∼	∼	∼	∼
	**Fruiting date**	13 May	14 May	13 May	13 May	13 May	14 May	14 May
	**Withering date**	21 May	21 May	22 May	22 May	22 May	22 May	22 May
	**Life cycle (d)**	224 ± 0.008^b^	224 ± 0.009^b^	225 ± 0.011^a^	225 ± 0.008^b^	225 ± 0.007^b^	225 ± 0.008^a^	225 ± 0.011^a^

**Notes.**

CK; W1, increase 30% in precipitation; W2, increase 50% in precipitation; N1, increase 3 g N m^−2^ yr^−1^; N2, increase 6 g N m^−2^ yr^−1^; precipitation plus nitrogen : W1N1, W1 + N1; W2N1, W2 + N1. Oct, October. Different lowercase letters indicate significant differences (*P* < 0.05) among increased precipitation and nitrogen and precipitation plus nitrogen.

### Plant traits

**Figure 2:**
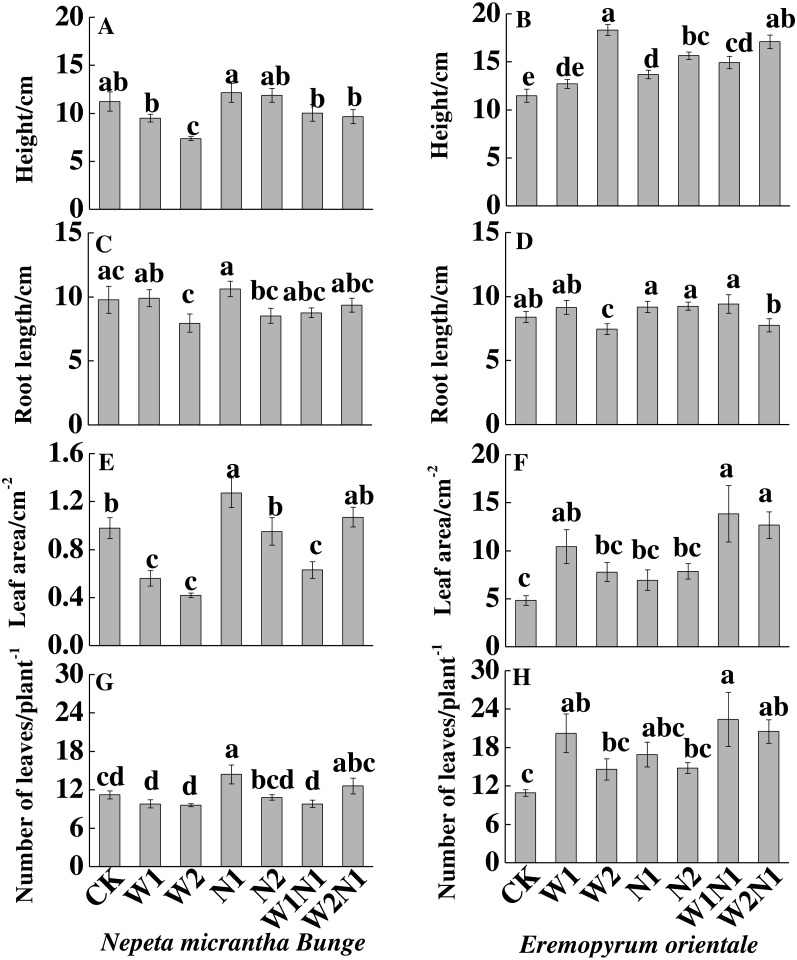
Effects of increased precipitation and nitrogen and precipitation plus nitrogen treatments on plant traits (mean ± 1s.e.) of* Nepeta micrantha* (A, C, E, G) and *Eremopyrum distans* (B, D, F, H). CK, control treatment; W1, increase 30% in precipitation; W2, increase 50% in precipitation; N1, increase 3 g nitrogen m^−2^ yr^−1^; N2, increase 6 g nitrogen m^−2^ yr^−1^; precipitation plus nitrogen: W1 + N1, W1N1; W2 + N1, W2N1. Different lowercase letters indicate significant differences among increased precipitation and nitrogen and precipitation plus nitrogen treatments.

Increased precipitation (W1 and W2) significantly decreased the height, root length and leaf area (*P* < 0.01) of *N. micrantha* but had nearly no effect on leaf number; Increased nitrogen and precipitation plus nitrogen have almost no effect on the height, root length and leaf number, but N1 significantly increased the leaf area and leaf number of *N. micrantha* (*P* < 0.01, Figs. 2A, 2C, 2E, 2G), and W1N1 significantly decreased the leaf area of *N. micrantha* (*P* < 0.01, Fig. 2E). Conversely, increased precipitation, increased nitrogen, and precipitation plus nitrogen treatments significantly increased the height, leaf area, leaf number of *E. distans* (*P* < 0.01), with the exception of W2 significantly inhibited the root length of *E. distans* (*P* < 0.01, Figs. 2B, 2D, 2F, 2H).

**Figure 3:**
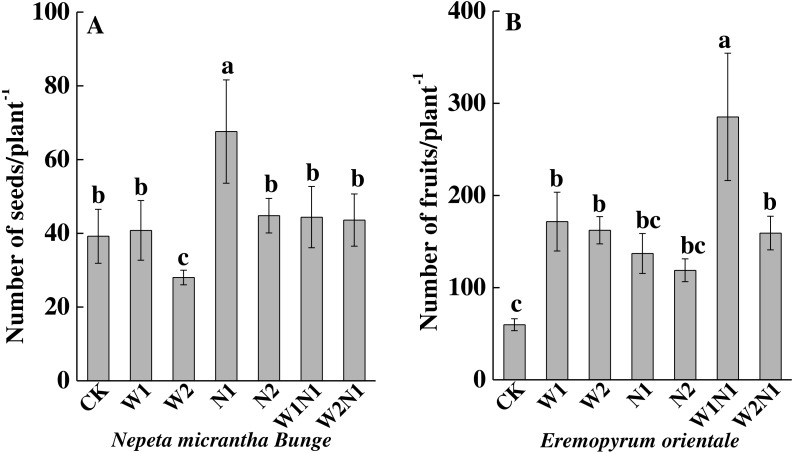
Effects of increased precipitation and nitrogen and precipitation plus nitrogen on seed number per plant (mean ± 1s.e) of *Nepeta micrantha* (A) and *Eremopyrum distans* (B). CK, control treatment; W1, increase 30% in precipitation; W2, increase 50% in precipitation; N1, increase 3 g nitrogen m^−2^ yr^−1^; N2, increase 6 g nitrogen m^−2^ yr^−1^; precipitation plus nitrogen: W1 + N1, W1N1; W2 + N1, W2N1. Different lowercase letters indicate significant differences among increased precipitation and nitrogen and precipitation plus nitrogen treatments.

To *N. micrantha*, W1, N2, W1N1 and W2N1 had nearly no effect on the seed number, but N1 significantly increased seed number (*P* < 0.01, Fig. 3A); however, W2 significantly reduced seed number (*P* < 0.01). To *E. distans*, increased precipitation, increased nitrogen and precipitation plus nitrogen significantly increased fruit number, and the increase of W1N1 in fruit number was the greatest (*P* < 0.01, Fig. 3B).

### Biomass accumulation and allocation

**Figure 4:**
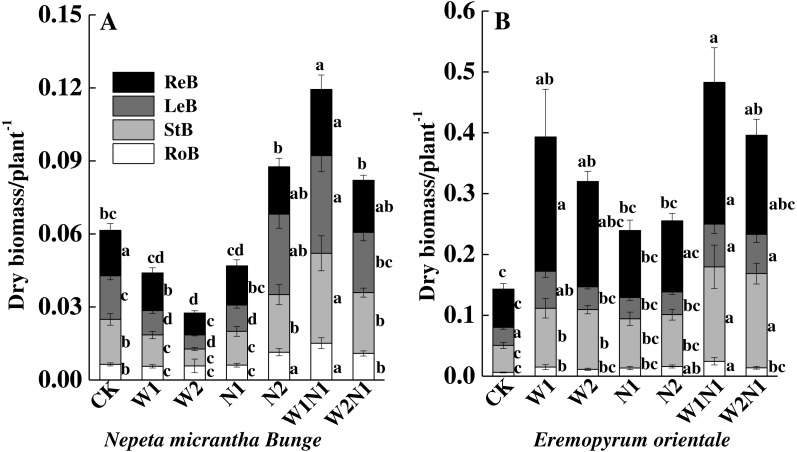
Effects of increased precipitation and nitrogen and precipitation plus nitrogen on dry mass accumulation (mean ± 1s.e) of *Nepeta micrantha* (A) and *Eremopyrum distans* (B). CK, control treatment; W1, increase 30% in precipitation; W2, increase 50% in precipitation; N1, increase 3 g nitrogen m^−2^ yr^−1^; N2, increase 6 g nitrogen m^−2^ yr^−1^; precipitation plus nitrogen: W1 + N1, W1N1; W2 + N1, W2N1. ReM, Reproduction mass; LeM, Leaf mass; StM, Stem mass; RoM, Root mass. Different lowercase letters indicate significant differences among increased precipitation and nitrogen and precipitation plus nitrogen treatments.

Increased precipitation (W1 and W2) decreased the total biomass of *N. micrantha* (*P* < 0.01, Fig. 4A), and the decrease of W2 is more significant than W1. The effect of N1 on the total biomass of *N. micrantha* is not significant (*P* = 0.98), but N2 significantly increased the total biomass of *N. micrantha* (*P* < 0.01), and precipitation plus nitrogen (W1N1 and W2N1) also significantly increased the total biomass of *N. micrantha* (*P* < 0.01, Fig. 4A). Increased precipitation, increased nitrogen and precipitation plus nitrogen significantly increased the total biomass of *E. distans*, and the increase of W1N1 in the total biomass is more significant than other treatments (*P* < 0.01, Fig. 4B).

**Figure 5:**
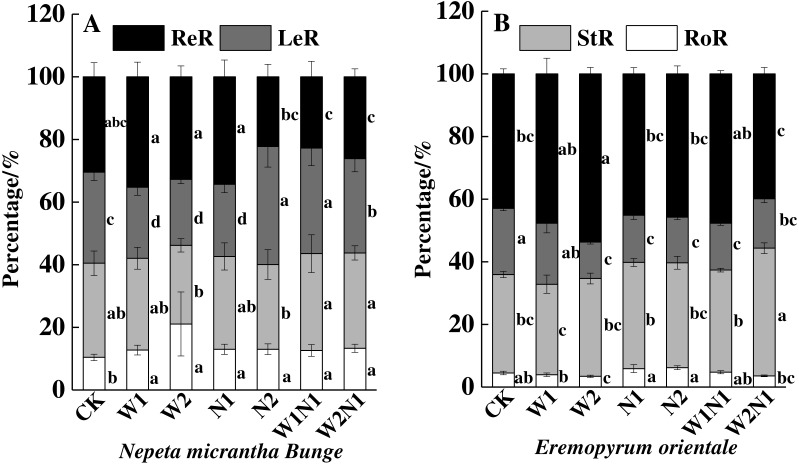
Effects of increased precipitation and nitrogen and precipitation plus nitrogen on biomass allocation (mean ± 1s.e) of *Nepeta micrantha* (A) and *Eremopyrum distans* (B). CK, control treatment; W1, increase 30% in precipitation; W2, increase 50% in precipitation; N1, increase 3 g nitrogen m^−2^ yr^−1^; N2, increase 6 g nitrogen m^−2^ yr^−1^; precipitation plus nitrogen: W1 + N1, W1N1; W2 + N1, W2N1. Different lowercase letters indicate significant differences among increased precipitation and nitrogen and precipitation plus nitrogen treatments.

The correlation analysis also revealed a positive correlation between plant traits (height, root length, leaf area, number of leaves, and number of seeds) and the total biomass accumulation of *E. distans* and *N. micrantha*, with the exception of the height of *N. micrantha* (Table 2). Increased precipitation, nitrogen, and precipitation plus nitrogen significantly increased the reproduction percentage of *E. distans* (*P* < 0.01), but N2, W1N1 and W2N1 slightly decreased the reproductive percentage of *N. micrantha* and W1, W2 and N1 slightly increased the reproductive percentage of *N. micrantha* (Fig. 5A). W1, W2 and N1 decreased the leaf percentage of *N. micrantha* (*P* < 0.01), but N2, W1N1 and W2N1 increased the leaf percentage of *N. micrantha* (*P* < 0.01). Increased precipitation, nitrogen, and precipitation plus nitrogen decreased the leaf percentage of *E. distans* (Fig. 5B). Increased precipitation, nitrogen, and precipitation plus nitrogen had little effect on the stem percentage of *N. micrantha* and *E. distans*, with the exception of precipitation plus nitrogen increased the stem percentage of *E. distans*. The root percentage of *N. micrantha* increased significantly with increased precipitation, nitrogen, and precipitation plus nitrogen (*P* < 0.01), and increased nitrogen increased the root percentage of *E. distans*, but W2 treatment decreased the root percentage of *E. distans*; however, the other treatments (W1, W1N1, and W2N1) had little effect on the root percentage of *E. distans*.

**Table 2:** Pearson correlations among life history traits of Nepeta micrantha and *Eremopyum orientale*.

		**He**	**Rl**	**Nl**	**Ns**	**La**	**Rom**	**Sm**	**Lm**	**Rem**	**Tm**
***Nepeta micrantha***	**He**	1									
	**Rl**	−.302^*^	1								
	**Nl**	0.097	.500^**^	1							
	**Ns**	0.217	.465^**^	.836^**^	1						
	**La**	.255^*^	.349^**^	.881^**^	.890^**^	1					
	**Rom**	0.121	.529^**^	.769^**^	.858^**^	.808^**^	1				
	**Sm**	.368^**^	.336^**^	.850^**^	.889^**^	.929^**^	.784^**^	1			
	**Lm**	0.165	.279^*^	.790^**^	.778^**^	.882^**^	.736^**^	.850^**^	1		
	**Rem**	0.145	.255^*^	.545^**^	.683^**^	.605^**^	.588^**^	.611^**^	.583^**^	1	
	**Tm**	0.233	.331^**^	.757^**^	.857^**^	.834^**^	.767^**^	.853^**^	.810^**^	.928^**^	1
***Eremopyum orientale***	**He**	1									
	**Rl**	0.015	1								
	**Nl**	.596^**^	0.182	1							
	**Ns**	.685^**^	.311^**^	.594^**^	1						
	**La**	.560^**^	.309^**^	.650^**^	.591^**^	1					
	**Rom**	.489^**^	.360^**^	.614^**^	.604^**^	.623^**^	1				
	**Sm**	.703^**^	.308^**^	.798^**^	.765^**^	.762^**^	.772^**^	1			
	**Lm**	.525^**^	0.216	.842^**^	.550^**^	.812^**^	.737^**^	.850^**^	1		
	**Rem**	.667^**^	.342^**^	.625^**^	.884^**^	.699^**^	.638^**^	.860^**^	.667^**^	1	
	**Tm**	.663^**^	.317^**^	.821^**^	.763^**^	.817^**^	.829^**^	.970^**^	.925^**^	.873^**^	1

**Notes.**

He height Rl root length Nl number of leaves Ns number of seeds La leaf area Rom root mass Sm stem mass Lm leaf mass Rem reproductive mass Tm total mass. N = 120

* *P* < 0.05.

** *P* < 0.01.

### Seed germination of the offspring

**Figure 6:**
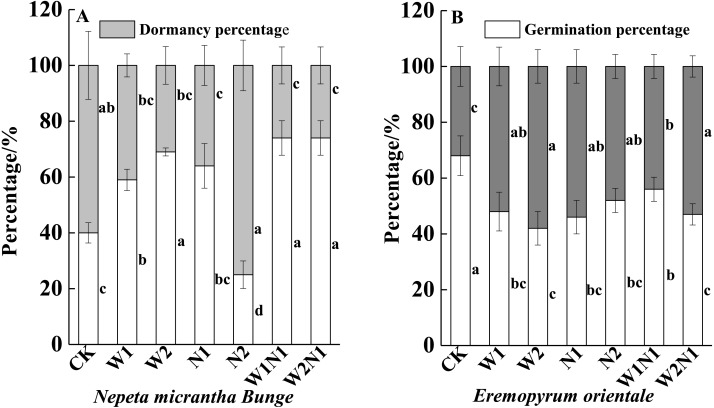
Effects of increased precipitation and nitrogen and precipitation plus nitrogen on offspring (seeds)germination (mean ± 1s.e) of *Nepeta micrantha* (A) and *Eremopyrum distans* (B). CK; W1, increase 30% in precipitation; W2, increase 50% in precipitation; N1, increase 3 g m^−2^ yr^−1^ in nitrogen; N2, increase 6 g m^−2^ yr^−1^ in nitrogen; precipitation plus nitrogen: W1N1, W1 + N1; W2N1, W2 + N1. Different lowercase letters indicate significant differences among increased precipitation and nitrogen and precipitation plus nitrogen treatments.

W1, W2, N1, W1N1 and W2N1 significantly increased the germination percentage of seeds produced by *N. micrantha* plants (*P* < 0.01), and the increase of W2 is more significant than W1, but N2 significantly reduced the germination percentage of seeds produced by *N. micrantha* plants (*P* < 0.01, Fig. 6A). Increased precipitation, nitrogen, and precipitation plus nitrogen significantly reduced the germination percentage of seeds produced by *E. distans* plants, and the reduce of W1N1 is more significant than W2N1 (*P* < 0.01, Fig. 6B).

## Discussion

Increased precipitation, nitrogen, and precipitation plus nitrogen prolonged the life cycle of both *N. micrantha* and *E. distans*, which allows plants to absorb water and nitrogen for a longer period of time and promoted vegetative growth (Cleland et al., 2006; Sherry et al., 2007; Han et al., 2016). Plant traits are external manifestations of plants and are easily affected by environmental factors (Jones, 1985). W2 significantly inhibited the root length of *N. micrantha* and *E. distans* compared to the control, this indicates that increased precipitation in the upper soil profile stimulated roots to stop growing into the deeper layers of soil. However, the relatively dry soil of the control promoted root growth into deeper soil. Studies on *Stipa bungeana* in the desertified grasslands of the Ordos Plateau in Inner Mongolia (China) also have found that root length corresponded to depth of water in the soil profile (Cheng et al., 2006). Thus, root is probably the sensitive organ to be affected by precipitation, and significant differences of plant traits in response to increased precipitation and nitrogen are most likely related to the roots (Eviner & Chapin, 2003; Griffin-Nolan et al., 2018). *E. distans* has a fibrous root system and a sand traps around the roots, it expanding the contact range between the root system and the soil (Verma et al., 2005), which are very beneficial for moisture-holding and nitrogen uptake (Wang & Wang, 2009). Thus, increased precipitation, nitrogen, and precipitation plus nitrogen significantly increased the height, leaf area, leaf number and seed number of *E. distans*. By contrast, *N. micrantha* has a taproot system consisting of a fixed main root and few lateral roots (Ding et al., 2016). Fibrous root systems are more efficient than taproot system at capturing mobile ions from spatially and temporally heterogeneous soils (Dunbabin, Rengel & Diggle, 2004). Therefore, most plant traits of *N. micrantha* showed no or negative response to increased precipitation. The reason is the loss of nitrogen caused by the increased precipitation. A previous study on soil nitrogen at the same field site also found that increased nitrogen leaching in the rhizosphere under increased precipitation (Huang et al., 2016). For *E. distans*, increased nitrogen and precipitation plus nitrogen treatments significantly increased the height, leaf area, leaf number and seed number, which also supports the role played by the roots of *E. distans* in strong water uptake and soil fertility retention. Thus, roots probably determine the difference of plants in response to environmental changes. Future increased precipitation and nitrogen deposition are likely to promote the growth of fibrous root system species, but it have a negative impact on taproot system species.

Biomass is the primary manifestation of plant energy accumulation (Eisenhauer & Scheu, 2008; Sherry et al., 2010)*.* A positive correlation between plant traits and biomass accumulation has been confirmed (Lu et al., 2014). In this study, the correlation analysis also revealed a positive correlation between plant traits and the total biomass accumulation of *N. micrantha* and *E. distans*, with the exception of the height of *E. distans*. The reason for the lack of correlation between the height and biomass accumulation is likely related to the growth characteristics of *E. distans*. We observed that plants of *E. distans* grow to a certain height (about 20 cm), and turn into growing branches (Y Chen, pers. obs., 2017), instead of increasing the height. Thus, the biomass accumulation of *E. distans* shows a nonsignificant correlation with the height. Our results also showed that the effect of N1 on the total biomass of *N. micrantha* is not significant, but N2 significantly increased the total biomass of *N. micrantha*. This is probably related to the nitrogen utilization percentage of *N. micrantha*. In the same study area, Cui et al.’s (2017) ^15^N tracer experiment found that total nitrogen recovery percentage of herb was <10%, for one species, the nitrogen utilization percentage is much lower. Thus, we speculated that the amount of nitrogen (N1) was too less to be utilized by *N. micrantha*, and only when the amount of nitrogen (N2) reached a certain amount can promote plant growth. Therefore, our results indicate that future increased nitrogen deposition will not have a significant impact on plant growth of species levels in the short term, whereas this promotion will be more significant with enhancing nitrogen deposition.

The biomass allocation reflects the response of plant to the environment (Sugiura, Kojima & Sakakibara, 2016). With increased precipitation, the root and reproductive percentage of *N. micrantha* showed an increasing trend. The possible explanation is that plants allocated more biomass to the root to facilitate water and nutrient uptake and allocated more biomass to reproductive organs to maintain the population. Similar conclusions have been drawn about *Ceratocarpus arenarius* and *Erodium oxyrrhynchum* under drought stress (Zhang, Sun & Tian, 2007). By contrast, increased nitrogen improves the poor soil and provides nutrients for plant growth, hence, *N. micrantha* allocated more biomass to vegetative organs (stems and leaves), in order to maximize the light capture. For *E. distans*, increased precipitation and nitrogen increased the total biomass accumulation, which is consistent with many species in the Gramineae, such as *Setarria viridis*, *Pennisetum centrasiaticum* (Mao et al., 2016), *Glyceria spiculosa* (Chen et al., 2016), and *Leymus chinensis* (Yu, Yan & Jie, 2011). Increased nitrogen reduced or eliminated the nitrogen limitation on the growth of *E. distans*, and it improved the light use efficiency, accelerated plant growth with sufficient precipitation, and then increased its dominance in the community (Borer et al., 2014; Rajaniemi, 2002). Therefore, in the context of climate change, increased nitrogen deposition is likely to promote plant growth, but different species showed a differential response to increased precipitation.

The seed stage is the most tolerant stage of life history to the environment, and dormancy is beneficial for allowing the seeds to germinate under suitable conditions (Qu & Huang, 2005). Previous research found that under appropriate environmental conditions, plants increased proportion of seeds remaining dormant but with high reproduction may prevent overcrowding during the following year (Katja & Martina, 2010; Tielborger & Petru, 2010). Our results showed that the number of *E. distans* seeds that remained dormant increased with increased precipitation and nitrogen. Thus, an increase in dormancy reduces not only the competition caused by siblings, but also the risk of population extinction via bet-hedging strategies (Venable, 2007), and this strategy has been a sensible practice throughout the course of evolution (Evans et al., 2007). However, we found that the number of *N. micrantha* seeds that remained dormant generally decreased with increased precipitation. This finding is consistent with the conclusion that the maternal plants produced more non-dormant seeds when faced with environmental stress (Huang et al., 2016), and it provides a competitive advantage for the next generations. In addition, the decrease of W2N1 in the germination percentage of seeds produced by *E. distans* plants is more significant than W1N1. This is mainly related to increased precipitation improved the utilization percentage of nitrogen by herb (Cui et al., 2017), and showed an increase in the dormancy of offspring. Thus, the ability of plants to sense environmental changes can be passed on to the next generation by seed dormancy, which in turn affects population dynamics in the following year.

## Conclusions

Ephemeral plants are a special group of plants that are extremely sensitive to environmental changes. Future increased precipitation and nitrogen deposition in the Gurbantunggut Desert may particularly affect the life history of ephemeral plants. *N. micrantha* and *E. distans* are two common ephemeral plants species in the study area. Most of the life history traits of these two species responded similarly to increased nitrogen and precipitation plus nitrogen but responded differently to increased precipitation. These differential responses are related primarily to species characteristics and the amount of precipitation and nitrogen in the experiment. In response to increased nitrogen and precipitation plus nitrogen, the *N. micrantha* and *E. distans* plants produced greater amounts of dormant seeds in conjunction high reproduction, which can also prevent overcrowding in the following year. However, the *N. micrantha* and *E. distans* plants produced more nondormant seeds in conjunction with increased precipitation, and these plants could occupy their habitat via the rapid seed germination in the future. Therefore, the differential responses of *N. micrantha* and *E. distans* to increased precipitation and nitrogen may affect their ecological niches in the community.

##  Supplemental Information

10.7717/peerj.6158/supp-1 Data S1 Raw data

10.7717/peerj.6158/supp-2 Table S1 Results(F-value) of one-way ANOVA testing the effects of increased precipitation,nitrogen, and precipitation plus nitrogen on life history traits Note: df, Degree of freedom; He, Height; Rl, Root length; La, Leaf area; Ln, leaf number; Sn, Seed number; Rom, Root mass; Sm, stem mass; Lm, Leaf mass; Rem, Reproduction mass; Tm, Total mass; RoP, Root percentage; StP, Stem percentage; LeP, Leaf percentage; Rep, Reproduction percentage. **, *P* < 0.01; ***, *P* < 0.001.

## Additional Information and Declarations

### Data Availability

The following information was supplied regarding data availability: The raw measurements are provided in the Table S1 and Data S1.
