# Supplementary material for: Life history responses of two ephemeral plant species to increased precipitation and nitrogen in the Gurbantunggut Desert
Source: PeerJ. 2019 Jan 11;7:e6158. doi: 10.7717/peerj.6158 (PMC6330950; doi:10.7717/peerj.6158)
Supplement: Table S1 — Note: df, Degree of freedom; He, Height; Rl, Root length; La, Leaf area; Ln, leaf number; Sn, Seed number; Rom, Root mass; Sm, stem mass; Lm, Leaf mass; Rem, Reproduction mass; Tm, Total mass; RoP, Root percentage; StP, Stem percentage; LeP, Leaf percentage; Rep, Reproduction percentage. **, P < 0.01; ***, P < 0.001. [file peerj-07-6158-s002.docx]

|  | **df** | **He** | **Rl** | **La** | **Ln** | **Sn** | **Rom** | **Sm** | **Lm** | **Rem** | **Tm** | **RoP** | **StP** | **LeP** | **ReP** |
| --- | --- | --- | --- | --- | --- | --- | --- | --- | --- | --- | --- | --- | --- | --- | --- |
| ***Nepeta micrantha*** | 6 | 4.341** | 4.785*** | 1.839 | 12.807*** | 1.723 | 5.424*** | 8.03*** | 12.157*** | 3.146** | 8.977*** | 0.946 | 2.274** | 9.738*** | 5.822*** |
| ***Eremopyrum orientale*** | 6 | 18.121*** | 2.547** | 3.053** | 4.437** | 4.672** | 2.856** | 5.194*** | 2.928** | 2.431** | 3.545** | 2.672** | 4.377** | 3.697** | 2.424** |
